# Polymers for Extrusion-Based 3D Printing of Pharmaceuticals: A Holistic Materials–Process Perspective

**DOI:** 10.3390/pharmaceutics12020124

**Published:** 2020-02-03

**Authors:** Mohammad A. Azad, Deborah Olawuni, Georgia Kimbell, Abu Zayed Md Badruddoza, Md. Shahadat Hossain, Tasnim Sultana

**Affiliations:** 1 Department of Chemical, Biological and Bioengineering, North Carolina A&T State University, Greensboro, NC 27411, USA; doolawuni@aggies.ncat.edu (D.O.); glkimbell@aggies.ncat.edu (G.K.); 2 Department of Chemical and Life Sciences Engineering, Virginia Commonwealth University, Richmond, VA 23284, USA; azmbadruddoza@vcu.edu; 3 Department of Engineering Technology, Queensborough Community College, City University of New York (CUNY), Bayside, NY 11364, USA; MSHossain@qcc.cuny.edu; 4 Department of Public Health, School of Arts and Sciences, Massachusetts College of Pharmacy and Health Sciences (MCPHS), Boston, MA 02115, USA; m0378698@stu.mcphs.edu

**Keywords:** polymers, pharmaceuticals, extrusion-based 3D printing, fused deposition modeling (FDM), pressure-assisted microsyringe (PAM), materials, process

## Abstract

Three dimensional (3D) printing as an advanced manufacturing technology is progressing to be established in the pharmaceutical industry to overcome the traditional manufacturing regime of 'one size fits for all'. Using 3D printing, it is possible to design and develop complex dosage forms that can be suitable for tuning drug release. Polymers are the key materials that are necessary for 3D printing. Among all 3D printing processes, extrusion-based (both fused deposition modeling (FDM) and pressure-assisted microsyringe (PAM)) 3D printing is well researched for pharmaceutical manufacturing. It is important to understand which polymers are suitable for extrusion-based 3D printing of pharmaceuticals and how their properties, as well as the behavior of polymer–active pharmaceutical ingredient (API) combinations, impact the printing process. Especially, understanding the rheology of the polymer and API–polymer mixtures is necessary for successful 3D printing of dosage forms or printed structures. This review has summarized a holistic materials–process perspective for polymers on extrusion-based 3D printing. The main focus herein will be both FDM and PAM 3D printing processes. It elaborates the discussion on the comparison of 3D printing with the traditional direct compression process, the necessity of rheology, and the characterization techniques required for the printed structure, drug, and excipients. The current technological challenges, regulatory aspects, and the direction toward which the technology is moving, especially for personalized pharmaceuticals and multi-drug printing, are also briefly discussed.

## 1. Introduction

Additive manufacturing, commonly known as three dimensional printing (3D printing), is seeing increased use in several different industries such as aerospace, motor vehicles, industrial machines, consumer products, electronics, military, medical, dental, etc. [1,2,3]. Prototyping, directly printed functional parts, patient-specific hearing aids, and dental restoration are a few examples where 3D printing contributes significantly [1]. 3D printing has also expanded to the pharmaceutical industry where it is used to manufacture pharmaceuticals (drug products, implants, drug delivery systems, etc.). 3D printing is especially advantageous in the manufacturing of personalized drug products, where current scale-focused industrial pharmaceutical processes fall short [1], which allows on-demand production of the drug product [4]. A drug product is comprised of an active pharmaceutical ingredient (API) and inactive functional excipients, which can be in the form of a solid dosage (pills, film, implantable devices, etc.) or as a liquid [5]. 3D printing has boomed in the pharmaceutical sector since the Food and Drug Administration’s (FDA) approval of the first 3D printed medicine, SPRITAM^®^, a levetiracetam drug manufactured by Aprecia Pharmaceuticals Company (USA) for the treatment of seizures [6].

Healthcare treatment has recently seen a movement toward personalized medicine. Ginsberg et al. define personalized medicine as medicine created from the analysis of an individual’s molecular profile. Progress in human genome research is quickly advancing its growth of personalized medicine [7]. This can be seen with the 2015 implementation of the Precision Medicine Initiative, an Obama administration (in the USA) national initiative focused on individualized care [8]. Current pharmaceutical manufacturing practices are not cost-effective for personalized medicine [2]. 3D printing pharmaceuticals are more suitable than current manufacturing practices for tailored solid dosages. 

**Figure 1:**
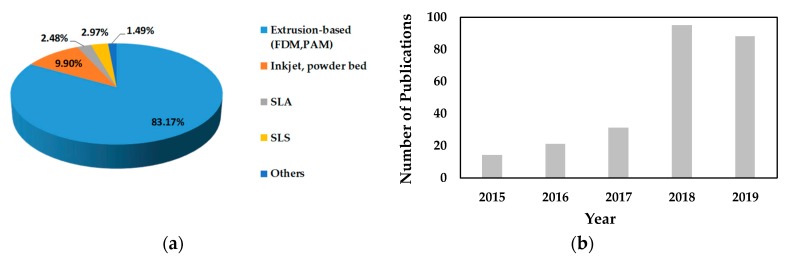
(**a**) The proportion of research articles published on different types of 3D printing processes in the last five years (2015-2019, total 202 articles); (**b**) The number of published scientific articles (research and review) in the period from 2015 to 2019 which reported the use of extrusion-based (fused deposition modeling (FDM) or pressure-assisted microsyringe (PAM)) 3D printing (source: Scopus database and PubMed).

3D printing was established by Sachs et al. in 1992 at Massachusetts Institute of Technology (MIT) [9,10]. Over the years, many different types of printing technologies have been developed [1,5,11]. The first wave of 3D printed pharmaceutical was fabricated using continuous inkjet printing. Wu et al. first used 3D printing in 1996 to make drug delivery devices [12]. Since this work, there have been significant advancements in 3D printing pharmaceuticals using inkjet printing as well as other printing technologies. The technologies used for drug product development are powder-based printing (powder bed and powder jetting) [13,14,15,16], extrusion (solid or semi-solid) based printing (fused deposition modelling (FDM), pressure-assisted microsyringes (PAM)) [17,18,19,20,21,22,23,24,25,26], stereolithographic (SLA) printing [27,28,29], selective laser sintering (SLS) printing [30,31,32], inkjet printing [33,34,35], digital light processing (DLP) [36,37], etc. Among all these printing processes, extrusion-based printing (FDM, PAM) has shown a growing interest among researchers due to the advantage of low cost, ability to fabricate hollow objects, ability to print using a range of polymers with or without drug, ability to tune drug release by tuning the geometry and polymer, and ability to print at room temperature (using PAM), etc. [38]. Figure 1a summarizes the published literature in the last five years on existing 3D printing technologies for pharmaceutical manufacturing and shows the proportion of research articles published on extrusion-based printing. Figure 1b presents the number of scientific publications over the last five years on extrusion-based 3D printing and shows the growing interest.

Each extrusion-based 3D printing technology requires certain material requirements in order for the successful printing of pharmaceuticals. The requirements are based on the nature of the printing process. To guarantee the successful printing of solid dosage pharmaceuticals, it is necessary to choose the appropriate polymers and other functional excipients besides the drugs [39]. Pharmaceuticals must also have proper criteria in order to meet FDA regulations. The selection of unsuitable polymers will result in unsatisfactory pharmaceuticals. Jones reviewed the different roles that polymers play specifically for solid dosage pharmaceuticals, i.e., tablets and caplets [40]. In tablets and caplets, polymers can be used as binders, disintegrants, compression aids, diluents, or fillers. Polymers can also be used for controlled drug release applications. Hence, polymers are used in the 3D printing of pharmaceuticals for different purposes such as to control the dosage shape, size, drug release, etc. Polymers’ multifaceted utilization in solid dosage drug delivery systems solidifies its importance in pharmaceutical 3D printing applications.

There are several review articles published on extrusion-based 3D printing [11,41,42,43,44,45,46,47,48,49,50,51]. However, those published articles focused on different aspects of this technology. For example, Araujo et al. discussed updates on FDM technology, its challenges, and how it can be integrated with the pharmaceutical production process [43]. Tan et al. discussed hot-melt extrusion, FDM 3D printing, and how both can be combined for advanced pharmaceutical applications [44]. Goole and Amighi discussed different 3D printing (including extrusion-based) processes and process parameters that can be controlled [11]. Kjar and Huang et al. discussed different 3D printing processes, then emphasized on micro-sized pharmaceutical applications [45]. Gioumouxouzis et al. discussed different dosage forms and devices that can be printed using a different 3D printing process [46]. Vithani et al. discussed potential opportunities for the 3D printing of soft materials such as lipids [48]. Joo et al. discussed different FDM process and parameters and discussed how FDM 3D printers can be used to control the release, make a novel dosage form, and deliver the customized doses [49]. Long et al. discussed appropriate polymers and key parameters required for FDM 3D printing and its usage in the printing of personalized tablets and drug delivery devices [50]. He et al. discussed FDM 3D printing methodology, suitable polymers, and important parameters that are required to print personalized tablets and drug delivery devices [51]. Konta et al. reviewed several 3D printing technologies and the polymers that are used successfully in those printing methods [52]. Unfortunately, there is no detailed discussion on how polymers should be selected based on materials characteristics, how their rheology impact on the printing process, and the required characterization for the 3D printed pharmaceuticals. The objective of this review is to discuss all these in detail to guide the researchers in selecting the right polymers and extrusion process to 3D print pharmaceuticals. In addition, a comparison between FDM and PAM 3D printing, the current challenges, regulatory aspects, and opportunities for extrusion-based printing are discussed.

## 2. Extrusion-Based 3D Printing

**Figure 2:**
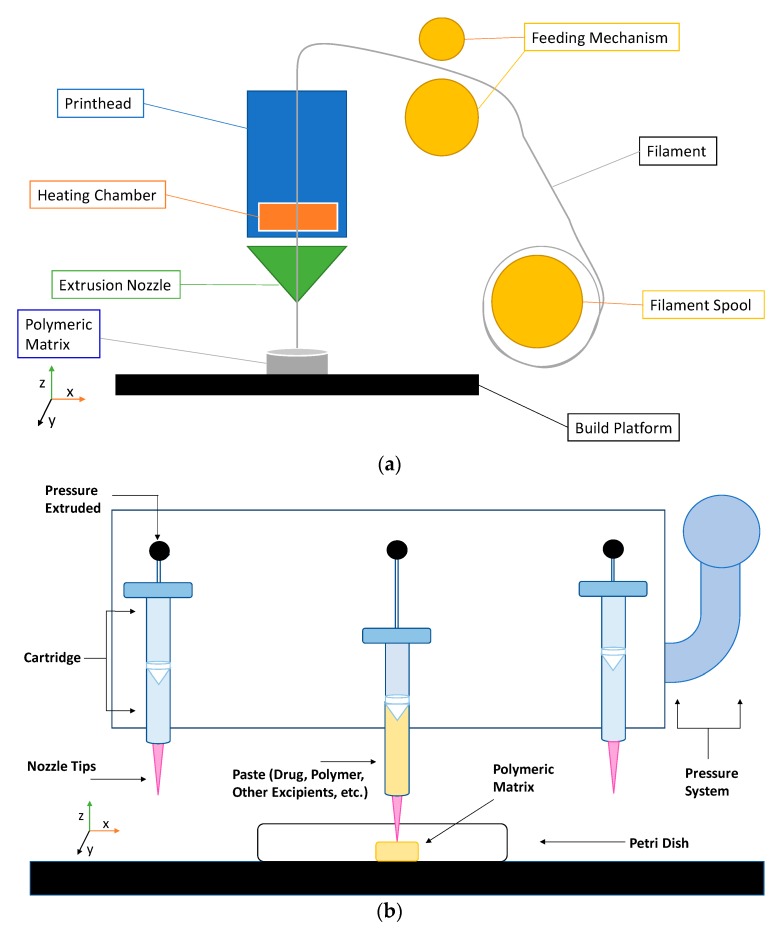
2-dimensional (2D) schematic of the extrusion-based 3D printing process: (**a**) fused deposition modeling (FDM), (**b**) pressure-assisted microsyringe (PAM).

Extrusion-based 3D printing can be categorized into two main types: Fused deposition modeling (FDM) and pressure-assisted microsyringe (PAM) [11]. Extrusion-based printing is also known as a nozzle-based deposition system [11]. It relies on a computer-controlled manufacturing method that deposit materials layer by layer through a nozzle to create a 3D structure with controlled composition and architecture. FDM also known as fused filament fabrication (FFF) is one of the most commonly used low-cost 3D printing techniques [53]. In FDM, the filament of the thermoplastic materials is melted or softened, then extruded from the printer’s head, and layer by layer deposited to form the 3D object (see Figure 2a). For pharmaceutical printing thermoplastic polymers have been used as a drug carrier and thermo-resistant drug molecules are used. Polyvinylpyrrolidone (PVP), polyvinyl alcohol (PVA), and polylactic acid (PLA) are the most commonly used pharmaceutical grade polymers for FDM [54]. The process parameters that must be controlled for FDM are the infill density, printer speed, layer height, and the temperature of the nozzle and build platform [19,20,38,55,56]. Prednisone, theophylline, etc. are examples of drug molecules [52]. The drugs can be loaded into the polymer filament by impregnation (known as FDMi) [11,38,56] or integrated with the polymer and make the filament by hot-melt extrusion (HME). If necessary additional functional excipients (e.g., plasticizer) are added during the filament making by HME [11]. Most recently, Pietrzak et al. showed the integration of HME and FDM in an attempt to increase the range of polymers that can be used to make filament for FDM [20]. Their study also showed that the drug loading can be increased to 50%.

Pressure assisted microsyringe (PAM) was used extensively in tissue engineering to create soft tissue scaffolds [11]. PAM has recently gained popularity in pharmaceutical applications. Semi-solids (gels or pastes) are extruded continuously layer-by-layer through a syringe based tool-head (Figure 2b). The extrusion is usually based on a pneumatic (pressured-air), mechanical, and solenoid piston, [17,57]. Semi solids contain an optimal mixture of polymer, solvent, and other functional excipients (if needed) having appropriate rheological properties that make it suitable for printing. PAM does not require high temperature whereas, drying as post-print processing is required. However, shrinking or deformation of the product may occur following the drying process. The printed object may also collapse if the deposited layer did not strengthen sufficiently to withstand the weight of the successive layers [53]. Nifedipine, Glipizide, etc. are drug examples that have been used in PAM [52]. It is also noted that for extrusion-based (FDM, PAM) 3D printing infill type, e.g., rectilinear, hexagonal, or honeycomb affect the mechanical strength (i.e., flexural strength) of the printed structure [58,59]. Table 1 provides a comparison between FDM and PAM 3D printing technologies [38,60,61,62,63]. 

**Figure 3:**
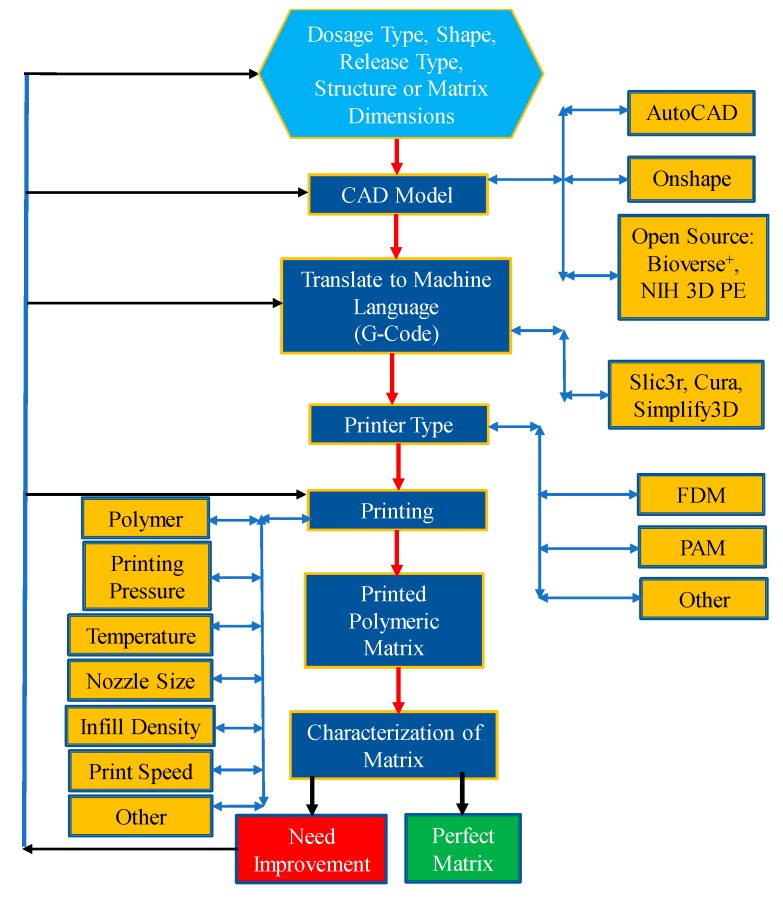
Workflow diagram or decision matrix for a 3D printing process. (CAD: computer-aided design, FDM: fused deposition modeling, PAM: pressure-assisted microsyringe, NIH 3D PE: National Institutes of Health 3D Print Exchange).

Figure 3 shows a workflow diagram or decision matrix that is required to consider from the beginning to the end of the printing process. The overall process starts with the decision making of dosage, types of application intended, then design using Computer-Aided Design (CAD), translating the design to machine language, deciding materials and process parameters, and finally printing the dosage. It is noted that printer resolution can also be a decision parameter when selecting the printer types. Due to the advances in technology standard 3D printing equipment now enable to achieve a print resolution of the order of a few hundred micrometers [64]. However, getting print resolution below a hundred micrometers is yet a major technical challenge [65]. 

**Figure 4:**
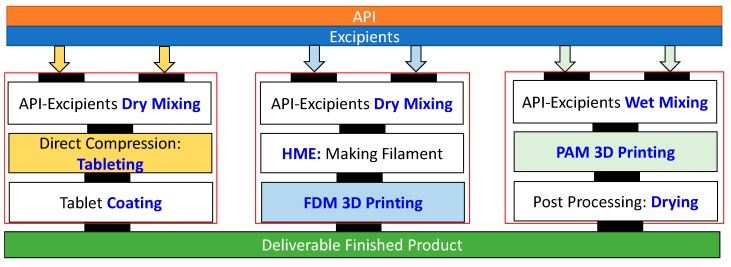
Comparison of different processing steps required for traditional direct compression (DC) tablet manufacturing vs. advanced manufacturing, 3D printing (FDM or PAM).

A comparison of processing steps required for traditional pharmaceutical manufacturing direct compressions (DC) vs. 3D printing (FDM and PAM) is shown in Figure 4. DC is considered the simplest manufacturing process for pharmaceutical tablets [66,67]. Both FDM and PAM require the same number of processing steps to manufacture deliverable finished products from raw materials (API and excipients). However, the added advantage for 3D printing is that it requires a smaller footprint, can be remotely controlled, and is not only for use with small batches but also for individual pills to be fabricated within a single batch of materials. These characteristics make 3D printing manufacturing closer to the patient’s specifications. The important aspects for 3D printing, similar to DC processing are that stable, reproducible starting materials need to be supplied.

## 3. Polymers Role on Extrusion-Based 3D Printing of Pharmaceuticals

Table 2 summarizes the materials (drugs, polymers, and other functional excipients) compositions used in recently published articles that utilized extrusion-based (either FDM or PAM) 3D printing for pharmaceuticals. Typically, in FDM the drug and polymer are mixed and made into filament by using HME (hot melt extrusion) and then extruded through the nozzle whereas, for PAM they are mixed in a solvent to make a paste. The semi-solid paste is then extruded through the nozzle. HME is used primarily for pharmaceutical solid dispersion manufacturing. It is a solvent-free process, employing heat and mechanical shear, and can be coupled with 3D printing [68,69]. Besides polymers, there are other functional excipients (plasticizer, insoluble filler, antioxidants, etc.) that are also used in either FDM or PAM 3D printing [60,69]. However, the polymer contains a large proportion of all ingredients. Hence, the polymer plays a critical role in forming the drug–polymer matrix suitable for dosage, and overall materials processability through process unit. This is applicable for both FDM and PAM. In this review, polymers are analyzed from both materials and process perspective. In the materials perspective (Section 3.1), polymers’ physicochemical properties, their suitability for the FDM or PAM printing process, dosage types, and types of drug release from the matrix are discussed. From the process perspective (Section 3.2), how these polymers impact the process operations are discussed.

### 3.1. Materials Perspective 

#### 3.1.1. Carbopol^®^

Carbopol^®^ homopolymers are high molecular weight, crosslinked polyacrylic acid polymers [78]. Crosslinking is done with allyl sucrose or allyl pentaerythritol. Polymers are synthesized in either ethyl acetate or cosolvent ethyl acetate/cyclohexane mixture. Carbopol^®^ 971P and 974P are suitable for PAM 3D printing. Carbopol^®^ 971P is a lightly crosslinked polymer having a viscosity of 4000–11,000 cP (0.5 wt% suspension), which will result in flow like honey in a semisolid formulation [78,79]. It is suitable for extended/controlled-release tablets, oral liquids and suspension [79]. Carbopol^®^ 974P is a highly crosslinked polymer and produces highly viscous gels [80]. The viscosity of 0.5 wt% suspension of Carbopol^®^ 974P is 29,400–39,400 cP [78]. It is suitable for extended-release tablet formulation.

#### 3.1.2. Ethylcellulose (EC)

Ethylcellulose (EC) is often used as a polymer in pharmaceuticals and has recently found use in 3D printed pharmaceuticals. It is a water-insoluble thermoplastic polymer. These properties associated with EC are taken advantage of its usage in FDM 3D printing in the pharmaceutical industry. As a polymer in drug formulations, it is often used for its sustained release capabilities [81]. EC must undergo some form of sample preparation, such as dissolution in acetone or the addition of a plasticizer before it can be used in FDM printing [82].

#### 3.1.3. Eudragit^®^

Eudragit^®^ polymers are a set of synthetic polymethacrylate used in pharmaceutical drug formulations. They are non-biodegradable, non-absorbable, nontoxic and amorphous polymer [83]. According to Evonik, all Eudragit polymers have thermoplastic properties, low glass transition temperatures (between 9 °C and > 150 °C), high thermostability, and high miscibility with APIs and other excipients [83,84]. Hence, they are suitable for hot-melt extrusion. Varying the functional group on the polymer dictates the type of drug release it is best suited for. For example, the Eudragit E series is for immediate release drugs. Eudragit^®^ E series is suitable for gastric fluid as it is soluble at lower pH up to pH-5. Eudragit^®^ L and S series show delayed release in drug formulations. The L and S series vary in pH. Eudragit^®^ RL and Eudgrait^®^ RS are used for time-controlled release purposes. These series are insoluble with pH-independent swelling [84]. Eudragit^®^ RL has high permeability while Eudragit^®^ RS has low permeability. Combining the series together enables pharmaceuticals with customized time-controlled release profiles. While it has been used successfully with FDM methods to create immediate-release tablets when used with a plasticizer, the 3D printing process was unreliable and the nozzle was frequently clogged [85].

#### 3.1.4. Hydroxypropyl Cellulose (HPC)

Hydroxypropyl cellulose (HPC) is a flexible, water-soluble polymer. HPC is made up of a monomer that is comprised of a glucose molecule with multiple hydroxypropyl substituents. HPC is available in different viscosity grades, making it suitable for formulating drugs with different release profiles. Overheating and rapid changes in temperature drastically affect the stability of HPC and its viscosity. HPC has a low glass transition temperature in the range of −25 °C to 0 °C as moisture varies from ~10% to 1%approximately [86]. It has high thermostability, making it suitable for processes that require melting and extrusion. The viscosity of HPC decreases as temperature increases, which in turn increases the release rate of the selected API. High molecular weight HPC, compared to its low molecular weight counterparts, exhibits high swellability that is suitable for controlled-release matrices [87]. 

#### 3.1.5. Hydroxypropyl Methylcellulose (HPMC)

Hydroxypropyl methylcellulose (HPMC) is a swellable, water-soluble polymer that enhances the sustained release capabilities of active ingredients in pharmaceuticals [88]. The high swellability of HPMC has significant effects on the release kinetics of pharmaceuticals [89]. HPMC E5 is used for immediate-release tablets and suitable for the PAM printing method [11]. Under high UV-light exposure, HPMC remains stable [90]. The glass transition temperature (*T_g_*) of HPMC is 170–198 °C [91]. When heated above certain temperatures, an aqueous solution composed of HPMC will gel out of the solution. The thermal gelation may impact drug stability in regards to 3D printing.

#### 3.1.6. Polycaprolactone (PCL)

Polycaprolactone (PCL) is a semi-crystalline, biocompatible polyester with a melting point of 55–60 °C and *T_g_* of −54°C [92,93]. It has a great organic solvent solubility. It is used for long-term implant delivery devices due to its very low in vivo degradation [92,94]. PCL is often blended or co-polymerized with PLLA (poly(l-lactic acid)), PDLLA (a racemic mixture of PLLA and PDLA (poly(d-lactic acid)), PLGA poly(lactic-*co*-glycolic acid), etc. to improve polymer erosion [94]. PCL is considered a good elastic biomaterial due to its low tensile strength (~0.023 GPa) and high elongation at breakage (4700%) [95]. 

#### 3.1.7. Polylactic Acid (PLA)

Polylactic acid (PLA) is an insoluble, synthetic biodegradable polymer [96,97]. It is the most extensively researched and utilized biodegradable aliphatic polyester. PLA is a thermoplastic, high-strength, and high modulus polymer [93,98]. It is non-toxic because its monomers can be made from the fermentation of sugar. The drug release of PLA encapsulated medicines can be influenced by the manipulation of PLA crystallinity degree and mechanical stability [98]. PLA is a very brittle material with less than 10% elongation at break, which limits its use in the application where plastic deformation is required at higher stress levels [98]. PLA has four forms as it possesses chiral molecules. Among them PLLA and PDLLA are promising for pharmaceutical applications. PLLA has a melting temperature of around 175 °C, a *T_g_* of 60–65 °C, and a mechanical strength of 4.8 GPa; whereas, PDLLA has a slightly lower *T_g_* of 55–60 °C and a mechanical strength of 1.9 GPa [94]. PLA can last up to three hours in acid, which is more suited to drugs that require a delayed release [99]. 

#### 3.1.8. Polyvinyl Alcohol (PVA)

Polyvinyl alcohol (PVA) is a biocompatible, swellable water-soluble synthetic polymer [100,101]. It is also a thermoplastic polymer [62], exhibiting a *T_g_* of 85 °C, melting point range of 180 (partially hydrolyzed) to 228 °C (fully hydrolyzed), and a partially hydrolyzed viscosity ranging from 3.4 mPa·s to 52 mPa·s [11,52]. It is widely used in FDM [102]. PVA is suitable for immediate release tablets as it dissolves more readily in hydrochloric acid [99]. However, controlled release can be achieved using PVA if the capsule is designed as a series of concentric circles to delay release [103].

#### 3.1.9. Polyvinylpyrrolidone (PVP)

Polyvinylpyrrolidone (PVP) is a water-soluble polymer [53]. It is also capable of solubilizing in other organic solvents. PVP’s solubility properties are attributed to its chemical structure, where it displays hydrophilic and hydrophobic components [104]. The chemical structure also yields hydrogen bonding of PVP. The hydrogen bonding causes interactions and the formation of complexes with low molecular weight compounds. The *T_g_* of PVP has a direct relationship with its molecular weight and it reaches a plateau at about 175 °C which corresponds to a molecular weight of 100,000 [104].

#### 3.1.10. Poly(Ethylene Glycol) (PEG)

PEG is a water-soluble, biocompatible, and amphiphilic polymer whose derivatives are used for a variety of applications. PEG is also known as polyethylene oxide (PEO) or polyoxyethylene (POE), depending on its molecular weight [105]. Polymers with Mw <100,000 are usually called PEGs, while higher molecular weight polymers are classified as PEOs [105]. PEGDA (PEG diacrylate) is a polymer that is a derivative of polyethylene glycol (PEG). To create PEGDA, an acrylic group is added to the terminal hydroxyl end group in PEG. The acrylic groups aid in its polymerization process where photopolymerization and other techniques are used [106]. PEGDA has better mechanical strength than PEG due to the formation of cross-link [94].

#### 3.1.11. Soluplus^®^

Soluplus^®^ is a polymeric solubilizer with an amphiphilic chemical structure. It is a graft copolymer composed of polyethylene glycol, polyvinyl acetate, and polyvinyl caprolactam. BASF designed this copolymer to solubilize APIs that are typically poorly soluble. It is also very suitable for HME because of its *T_g_* of about 70 °C and low hygroscopicity [107].

**Figure 5:**
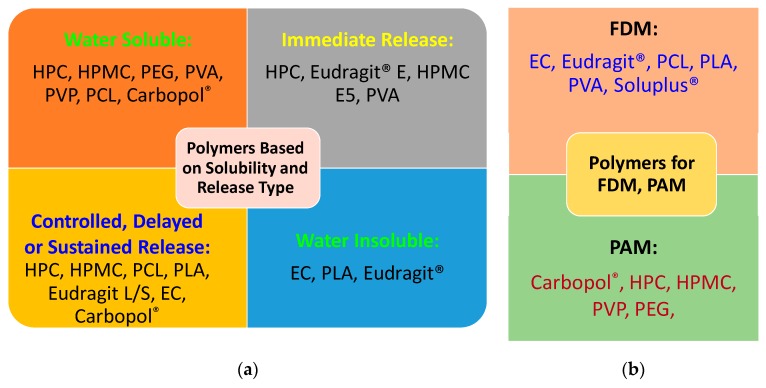
Summary of polymers based on (**a**) water solubility and drug release type, (**b**) their selection for either FDM or PAM 3D printing method.

In summary, all the polymers available are classified based on water solubility and the types of drug release which have been summarized in Figure 5a. Figure 5b shows their suitability to use for either FDM or PAM 3D printing process.

### 3.2. Process Perspective 

In the FDM 3D printing process, melted materials are used in the creation of products. This printing method requires constructing the material into printable filament and passing the filament through a heated nozzle [52]. In most experiments, filaments are either bought or created using HME. Printable filaments must have adequate rheological properties and mechanical strength to ensure proper processability in FDM 3D printing [52,93]. Goyanes et al. used commercially available PVA filaments impregnated with drugs to 3D printed pharmaceutical tablets. There was no indication that the rheological properties of the commercial PVA filaments changed. Therefore, there were no issues with processing the filaments through the printer [19,38]. In other studies, Goyanes et al. used HME to mix commercial PVA filament with active pharmaceutical ingredients. The mixture of PVA and API is extruded through a single screw extruder with a diameter of 1.75 mm at a screw speed of 15 rpm and a temperature of 170 °C. The filaments were able to be processed through the printer successfully. Goyanes et al. noted that the HME filaments were not significantly different from the commercial PVA filaments relative to the physical appearance, size, and mechanical behavior. The study did not include any rheological testing done on the created filaments [100,108]. 

The thermoplastic properties of a filament produced from HME may be affected if the filament has a high drug to polymer ratio [11]. If the drug-loading percentages are to be increased, a plasticizer may need to be added in order to soften the filament to suitable flexibility for printing [55]. Aho et al. described that soluble APIs can act as plasticizers when mixed with polymers during HME [109]. This leads to the mixture’s melt/glass transition temperature and viscosity decreasing. Yang et al. found a similar result and showed that when ibuprofen is the active ingredient, it acted as a plasticizer for the HME filaments, decreasing the stiffness (resistance to deflection or deformation by an applied force) of the filaments [110]. Yang et al. used a tensile test to determine the stiffness of the Ethyl Cellulose filaments and found a linear relationship between ibuprofen content and stiffness [110].

Stiffness and brittleness (and viscosity) are important properties to determine whether a filament will be suitable for FDM processing. Stiffness is typically defined using the ratio of load and deformation [111].

(1) $$Stiffness=\frac{Load}{Deformation}\;$$

Zhang et al. used breaking stress as load and breaking distance as deformation [71]. They used the 3-point bending flexural test to measure the stiffness and brittleness. In the study, the filaments were produced solely with EC and the model drug. They have suitable stiffness but are too brittle for FDM. The HPMC filaments created high stiffness and toughness but had low processability because of high melt viscosities and rough surfaces. The HPC LF and the HPC EF filaments are too soft and flexible to be processed by the FDM 3D printer. Soluplus^®^ and Eudragit^®^ L100 melt are unable to form HME extruded filaments at high temperatures, such as 140°C [71]. Okwuosa et al. used HME to create PVA filaments for 3D printing of pharmaceuticals. During the printing process, the PVA filaments exhibited poor flow from the nozzle in the FDM printer and could not form a stable structure. It was necessary to add talc to the filament as a thermostable filler [112]. Filaments composed of different combinations of polymers, created with HME, exhibited better mechanical and rheological properties suitable for FDM 3D printing compared to the individual polymer formulations [71].

Viscosity, an important rheological property, is largely dependent on temperature [109]. In FDM 3D printing, the recommended temperatures for printing are typically too high for printing pharmaceuticals. In a study by Pietrazk et al., a lower printing temperature was used in order to avoid thermal degradation of the API and the polymer [20]. At lower than optimal temperatures, the filament can block the nozzle when attempting to print because of increased viscosity. Similarly, in another study by Yang et al., low printing temperatures had an increase in the viscosity of the EC tablets which caused nozzle blockage and low bond strength between layers [110]. 

PAM printing eliminates the need for prior formulations of filament that is necessary for FDM. The materials extruded during PAM printing should be in a semi-solid form, also referred to as a paste or gel. These are typically formed by mixing polymer(s), functional excipient(s), and drug with an appropriate solvent(s) at a ratio that results in a paste suitable for printing [53]. Optimal paste should have suitable rheological properties to enable it to process through the printing system. These properties include viscosity, yield stress under shear and compression, and viscoelastic properties [113]. Rattanakit et al. developed an extrusion printer with the purpose of printing a Dexamethasone-21-phosphate disodium salt (Dex21P) tablet encapsulated by PLGA and PVA [114]. In this extrusion system, an air pressure line is connected to dispensing the paste. Paste with varying molecular weights of PVA is used for printing. The viscosities of the different ink solutions are measured and deemed suitable viscosities for printing, with values between 16.4 cP and 861.9 cP. As the molecular weight of PVA in the paste increases, the amount of air pressure for extrusion increases. For the solution with a viscosity of 16.4cP, an air pressure line was not necessary for extrusion. Rheological parameters of the paste must be configured to the needle of the print head for successful extrusion. 

Khaled et al. produced various pharmaceuticals using PAM printing. In 2015, Khaled et al. created a three-drug (multi-active) tablet [76]. To ensure that the print head nozzle does not experience blockage, they made the paste smooth and homogeneous. The proper flowability of the paste ensures that the printing process is successful. HPMC was used for the sustained-release compartments of the tablet. To keep the HPMC at an optimal viscosity for printing, hydroalcoholic gel, instead of water, was used as the binder. In another study Khaled et al. created a polypill with five different drug doses, using PAM printing [18]. HPMC and cellulose acetate are chosen as the polymers for the shell and sustained release compartments, respectively. To prevent nozzle blockage, both polymers were mixed with solvents to be made into a smooth and homogeneous paste. Khaled et al. state that if a polymer can be processed into a powder form, it can be printed using PAM printing [17]. A detail on polymer rheology and its impact on structure and process are elaborated in the next section.

## 4. Polymers Rheology and Its Impact on Structure and Process

**Figure 6:**
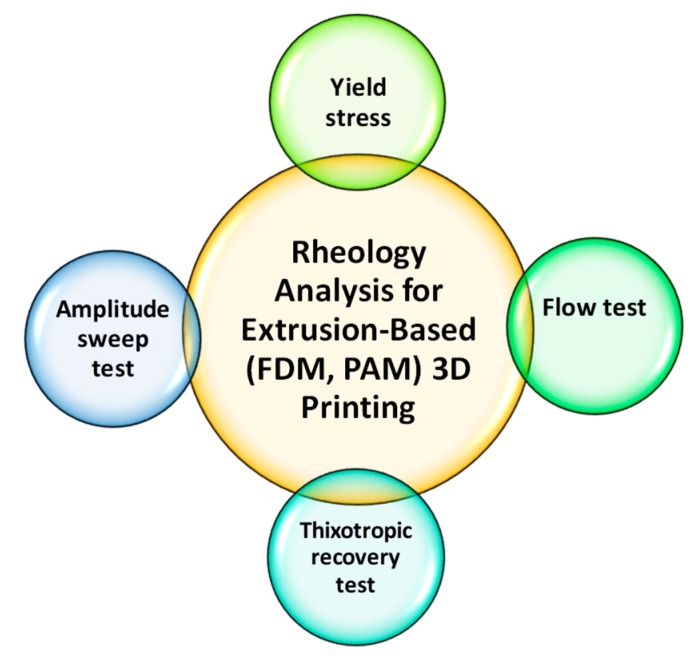
Important rheological tests are required for polymer–drug paste or molten dispersions to ensure their suitability and processability for extrusion-based (FDM, PAM) 3D printing.

Rheological properties of the polymers and polymer–API mixture play a vital role in predicting the processability of FDM and PAM 3D printing and the properties of the final pharmaceutical products (solid dosages) such as drug release. These rheological properties that depend on types of materials, i.e., polymers, excipients and formulation compositions are influenced by the nozzle diameter, pressure drop and feed rate, as well as the thermal properties of the feed, such as specific heat capacity, thermal conductivity, density and glass transition temperature [109,115]. The knowledge and better understanding of how the flow behavior of the feed materials changes as a function of time, shear and/or extensional deformation, and deformation rate, is of great help in tailoring the process conditions and choosing suitable polymer-carriers for melt processing by FDM and paste or gel processing by PAM 3D printing. Moreover, the addition of solid matters such as APIs, plasticizers, non-melting fillers particularly at high content in the polymer melts dramatically influences the flow properties of the pharmaceutical formulations, and therefore adjustment of the processing parameters, e.g., the extrusion temperature or nozzle speed is required [96,115,116]. The rheological properties are also considered as the major parameter that controls the reproducibility of the printed structure. Of particular concern to PAM 3D printing method is the polymer’s response to being extruded, how well it is able to adhere to previously printed layers, and its ability to hold the weight of subsequent layers [60]. Hence, the polymer–drug paste should be characterized by suitable apparent viscosity, viscoelastic properties, and yield stress under shearing and compression to be smooth and homogeneous to avoid nozzle blockage [113]. Figure 6 shows important rheological tests are required for the polymer–drug melts or paste to use it for FDM or PAM 3D printing. Aho et al. summarize the various rheological tools and measurement techniques that can be used to evaluate the flow behavior and processability of polymers and APIs with HME or FDM processing [109]. Hence, for extrusion based-3D printing, rheological measurements should be considered as a key part of the basic physicochemical characterization toolbox when selecting suitable polymer candidates, yet it remains underutilized [109].

The viscosity is the most important rheological parameter that plays a major role in determining optimal processing conditions for FDM and PAM 3D printing. It describes the resistance to flow and is given as a relationship of stress to the deformation rate. In the FDM process, a different force would be applied to the filament as it is pushed down by the drive gear and directs the heated filament toward the nozzle, where they are subjected to different shear rates. The shear rate at the printer liquefier depends on its dimensions and the printing speed, and because of the narrow nozzle diameter, it is typically very high [96]. Both temperature and shear rate affect melt viscosity, flow, and deformation behavior of materials during melt extrusion. The flow behavior index measures the degree of non-Newtonian characteristics for a material. The more the index deviates from 1, the more non-Newtonian its character becomes and the more viscosity is affected by strain. If a fluid has a flow behavior exponent less than 1, it is shear-thinning and exhibits more non-Newtonian characteristics [117]. The changes in viscosity of polymers can be associated with a shear-thinning behavior; whereas the viscosity of polymer can significantly be reduced when a higher shear rate is exerted during the printing process. A fluid that is shear-thinning is favored in PAM or FDM applications as the ‘shear-thinning’ property influences not only the capability to be pushed through a narrow nozzle at a given temperature but also the ability to regain structure and shape after deposition. Knowing the ideal viscosity range can help in predicting whether the new melt or paste formulation is extrudable. The viscosity has to be much lower than that for melt or paste extrusion for efficient printing and, at the same time, it should not be so low that it flows as liquids or very soft filaments from the printing nozzle. Viscosity data at high shear rates are more representative of the materials’ rheological properties during extrusion [118]. However, very few published works are available on the evaluation of the flow behavior by considering the process shear rates and access to the printability window of the FDM or PAM process. Nevertheless, the actual viscosity of the polymers during FDM is unable to be measured; and therefore, to date, no indicator or acceptable viscosity range can be provided to predict the success of the FDM process [119]. A straightforward approach is to compare the viscosity of the new formulation to that of a successfully extruded formulation (e.g., a commercial filament) using a rheometer. A dissimilar viscosity profile may not necessarily equate to an unextrudable melt, provided that they possess comparable viscosity at the operating shear rate; hence the shear rate of interest will need to be identified.

In order to estimate the feed filament viscosity in the 3D printing process, the volume flow rate through the printer nozzle was calculated from the pre-set nozzle speed, i.e., the speed at which molten material is deposited/extruded from the nozzle. The volume flow rate, Q can be calculated using the radius of the nozzle exit, r and the speed of extrusion v (i.e., printing speed):

(2) $$Q=\pi r^{2}v\;$$

The corresponding apparent shear rate at the nozzle wall can be semi-empirically determined using the following equation:

(3) $${\dot{\gamma }}_{app}=\frac{4Q}{\pi r^{3}}\;$$

For example, a printing speed (v) of 50 mm/s and a printer nozzle radius (r) of 0.1 mm equates to a flow rate of 6.3 mm^3^/s and consequently an apparent shear rate $({\dot{\gamma }}_{app}$) of ~1000 s^−1^. Due to the flow instabilities and sample rupture for high-viscosity fluids like polymer melts, measuring steady-state shear viscosity at the range of high shear rate is not possible by rotational rheometers [120], thus capillary rheometers should be used to access a high shear rate regime. Using the small amplitude oscillatory shear (SAOS) measurements and applying the Cox–Merz rule [121] which links together the dynamic and steady-state rheological properties, one can make estimations of the higher shear rate range too, albeit this is also limited to shear rates below 700 s^−1^ [109]. If the Cox–Merz rule is applicable, the complex viscosity from SAOS test and steady shear viscosity can be correlated to each other:

(4) $$\eta \left(\dot{\gamma }\right)=|\eta^{*}\left(\omega \right)|,\;\mathrm{when}\;\dot{\gamma }=\omega \;$$

In other words, if the Cox–Merz rule is valid, one can directly compare the complex viscosity vs. angular frequency results to shear viscosity vs. shear rate. A good overlapping between the results measured in SAOS and steady-state rotation shear (SSRS) was noticed for the green PLA, whilst the Cox–Merz rule failed for the white PLA [122]. The viscoelastic data shows that the typical Maxwellian behavior that is expected for pure polymers (storage modulus, G′ ~ $\omega^{2}$ and loss modulus, G″ ~ $\omega^{1}$ at low frequency) was observed for the green PLA, which behaved similarly to a viscous liquid with negligible elasticity. Storage modulus (G′) and loss modulus (G″) are measure of elastic response and viscous response of a polymer, respectively. The white samples, on the other hand, exhibited a marked elasticity and a much weaker ω-dependence of the moduli. The printing quality of these samples with the FDM technique could be correlated to the viscoelastic properties outside the printing apparatus [122]. Usually, amplitude sweep test or dynamic frequency sweeps or dynamic mechanical analysis is commonly used to measure the viscoelastic properties of polymer melts or paste such as storage modulus (G′), loss modulus (G″), and yield stress (*σ*).The crossover point (G′ = G″) provides information on the solid- or viscous-like behavior of the materials at various temperatures [118,123]. Its measurement essentially indicates how well the material withstands oscillation stress and how quickly it will break down. The higher the storage modulus, the more rigid the sample, and the more difficult it is to begin flow from the nozzle of a PAM printer [117]. This can lead to the nozzle becoming clogged before printing even begins, though after printing it will be the most rigid structure. Conversely, if this indicator is too low, the polymer will fall apart and not withstand oscillation. If G″ is greater than G′ at all frequency investigated, the polymer solution behaves and flows like a viscous material, and the printed material will generally retain its shape [124]. Yield stress is the point at which storage and loss modulus meet and is indicative of the amount of force required to make a material flowable, or transition from a solid to a liquid-like character. In PAM printing, this corresponds to the moment that the polymer is extruded through the nozzle. Therefore, the polymer should not have so great a storage modulus that it cannot be extruded and becomes clogged, but the loss modulus must not be so low as to allow the material to drain freely from the nozzle or fail to hold its shape. The yield stress of the polymer should be adequate for use on a standard PAM printer at a reasonable printing pressure [60]. A yield stress fluid is also desirable for its structural characteristics [124]. Thixotropic properties of the polymers which relate to the ability of a material to recover after the application of shear, are also important during PAM or FDM extrusion. Ideally, the polymer should be able to recover completely to its original state after a shear force is applied during extrusion, and as quickly as possible [125]. Recovery time is also important so that when the polymer is deposited on the printing platform, it can be given the time it needs to recover before any subsequent layers are added [77]. 

Controlling the dose of 3D printed tablets is also a challenge in single head FDM 3D printing. Previously commercially available filaments composed of widely used polymers such as polyvinyl alcohol (PVA) [19,56,126,127] in addition to newer ones such as Eudragit^®^ RL, Kollicoat^®^, IR and Soluplus^®^ [54] have been explored to make drug-loaded tablets by FDM 3D printing. All those studies highlighted several challenges involved in employing the printing techniques for pharmaceutical applications. The use of elevated temperatures (185–220 °C) and limited drug loading (0.063–9.5% *w*/*w*) renders it less suitable for many drugs particularly thermo-labile ones. HME has been used to compound high drug-loaded filaments as a feed for FDM 3D printing [61,62,71,128,129,130,131,132]. Tuning drug loading in such filaments, however, would significantly impact the plasticity as well as drug release profiles [82,128]. Although FDM is an extension of HME, the mode of shearing differs between the two technologies, and thus viscoelastic properties suitable for HME may not be suitable for FDM. As the FDM 3D printing process is particularly sensitive to changes in plasticity and rheological properties of the filament, it is therefore of paramount importance to craft compatible filaments so the printer can fabricate structures of a similar release profile from a wide range of doses. Recent studies have linked a filament’s 3D printing compatibility with the rheological properties of the backbone polymers used in FDM 3D printing process: polymethacrylate [62,132], PLA [96], polyvinylpyrrolidone-vinyl acetate (PVP-VA) [133], PCL [134], and polyethylene oxide (PEO)-PEG [135]. Pharmaceutical literature presents some examples of how rheology can be used in the FDM 3D printing process to rationalize the effect of melt behavior on printing quality, and Table 3 summarizes the polymers (excipients) and APIs, and the rheological analysis tools reported in the literature for extrusion-based (FDM, PAM) 3D printing.

**Figure 7:**
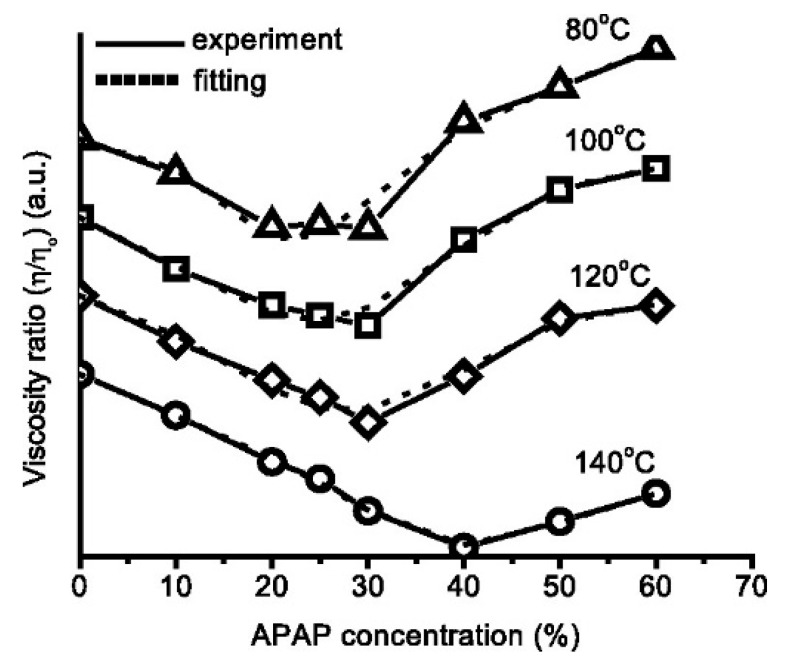
Viscosity ratio (*η*/*η_o_*) of acetaminophen (APAP) in poly (ethylene oxide) (PEO) at different temperatures to determine the solubility of the drug. Reprinted with permission from M Yang, International Journal of Pharmaceutics, Published by Elsevier, 2011 [123].

The miscibility between polymers and other materials in the blends can significantly affect the rheological properties of the systems which in turn impacts the processability and the qualities of 3D printed pharmaceuticals and also their performance. Recent studies by Sadia et al. showed that the complex viscosity of Eudragit EPO was ~8750 Pa·s at the FDM 3D printing temperature [132]. Upon compounding into filament via HME, the complex viscosity was dropped to approximately 4000 Pa·s at 1 rad/s angular frequency (with the 0% hydrochlorothiazide (HCT) drug). This drop-in viscosity could be linked to the reduced *T_g_* in these filaments (with the addition of plasticizer) and the decreased polymeric chain interaction. In a previous study, they reported that the introduction of a non-melting filler (tribasic calcium phosphate, TCP) can enhance the viscoelastic behavior in the system [62]. Replacing a non-melting filler (TCP) with an equivalent amount of low miscibility drug, HCT at a wide range of percentages (2.5–50%) resulted in comparable rheological behaviors with a predominantly viscous character with G′ < G″. However, when a drug with high miscibility with the polymer is incorporated in the filament, the complex viscosity dropped significantly (~743 Pa·s at 1 rad/s angular frequency). This illustrates the importance of drug miscibility with the polymer on the rheological performance of the filament in FDM 3D printing. Alhijjaj et al. explored the use of polymer blends as a formulation strategy to overcome the processability issues associated with commercially available FDM printers and to provide adjustable drug release rates from the printed dispersions [22]. The solid dispersions of felodipine with Eudragit^®^ and Soluplus^®^ were prepared using FDM printing after blending with plasticizers—PEG, PEO and Tween 80. The results demonstrated that the interplay between the miscibility of the excipients in the blends and the solubility of the polymer in the media can be used to manipulate the drug release rates of the dispersions. Many miscible/ soluble small-molecule APIs act also as plasticizers when mixed with polymers, lowering the molecular friction between the long, entangled polymer chains, which leads to decreasing of the melting/glass transition temperature and viscosity of the drug-polymer systems [109,118,137,138]. The solubility of a drug substance in the polymer matrix can be measured by a rheological method suggested by Suwardie and Yang [118,123]. The curves of the viscosity of a drug-polymer mixture vs. the drug loading generally have a ‘V’ shape, and the minimal point gives the drug’s solubility in the polymeric excipient as shown in Figure 7. If the API does not dissolve into the polymer matrix, it will form a solid dispersion, and the drug particles can be considered to behave the same way as inorganic filler particles in polymers. At high filler content, some particles are believed to form networks whose breaking down requires a certain amount of stress, yield stress, which causes a significant increase in viscosity at low deformation rates [62]. Both the plasticization by the dissolved drug and the presence of solid drug particles have implications on the melt processes such as hot melt extrusion or FDM 3D printing of concentrated mixtures [140]. The plasticization potentially enables the production of dosage forms with a high drug load using a low processing temperature and therefore avoiding thermal degradation of the drug [61,138].

**Figure 8:**
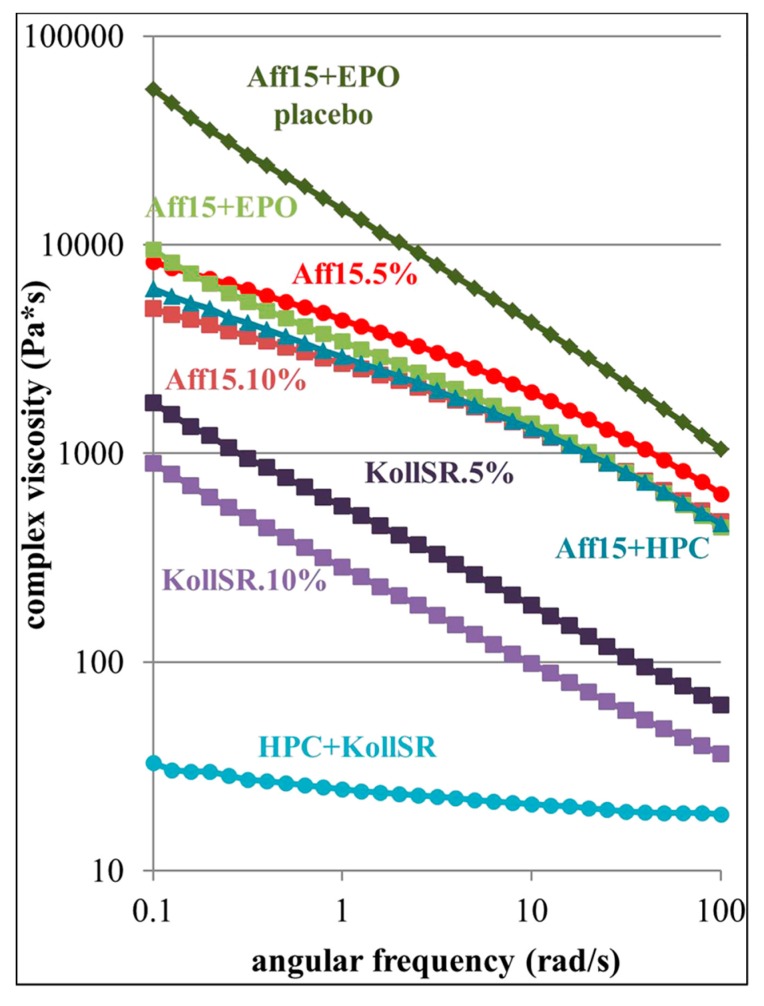
The rheological characterization of the blends of different polymers [Affinisol 15LV (Aff15), Kollidon SR (KollSR), Eudragit EPO (EPO), hydroxypropyl cellulose (HPC)]. Constituent effect visualized with frequency sweep at the maximum processing temperature (200 °C). Reprinted with permission from K Ilyés, European Journal of Pharmaceutical Sciences, Published by Elsevier, 2019 [136].

The selection of the FDM 3D printing process parameters such as extrusion temperature, shear rate requires the knowledge of the rheological properties of feedstock materials. The extrusion temperature should be set such that the complex viscosity falls within this range. It was reported that certain polymers, such as Affinisol™ 15 LV, can undergo shear-thinning during the extrusion process, which further reduces melt viscosity and facilitates melt extrusion at a lower temperature [141]. Recently Solanki et al. attempted to identify pharmaceutically acceptable polymers for the formulation of 3D printed tablets by FDM that would provide relatively rapid drug release [137]. They studied the rheological properties, i.e., complex viscosity of individual polymers, polymer–polymer binary mixture, drug–polymer binary mixtures, and drug–polymer–polymer ternary mixtures to rationalize their viscosities on FDM 3D printability. K. Ilyés et al. also reported the assessment of the printability window of different filaments based on pharmaceutically relevant polymer blends from the rheo-mechanical properties standpoint [136]. Each component in the blends modifies the rheology of the polymer matrix as shown in Figure 8, improving the shear thinning behavior and positively influencing the FDM processability window. Measuring G′, G″ and the crossover point (G′ = G″) for polymer–drug mixtures at different concentration and temperature also provides an idea to select the processing temperature during FDM 3D printing. In a study by Suwardie et al., the extrusion temperatures for acetaminophen-PEO system were selected between 120 °C and 140 °C as the mixture samples do not show crossover points at 120 °C and 140 °C within the tested frequency, and G″ is always higher than G′ in the temperature range, suggesting the viscous behavior of the mixture [118,123]. The molecular weight (MW) and molecular weight distribution (MWD) of the polymers can affect their printability because of their variable flow behaviour [116]. Increasing polydispersity (broader MWD) broadens the glass transition area and melting endotherm peak (for semi-crystalline polymers) observed in the DSC, as well as the transition area from the Newtonian plateau to the shear-thinning area in the viscosity curve profile [109]. Isreb et al. recently studied the effect of PEO of different molecular weight on the compatibility of HME compounded filaments for FDM 3D printing and their rheological properties to rationalize the effect of melt behaviors on their printability [135]. A lower molecular weight of PEO (100–200 K) yielded mechanically incompatible HME compounded filaments and a larger molecular weight of PEO (900 K) contributed to significantly high complex viscosity and inhibited material flow at a printing temperature of 110 °C and 145 °C. The molecular weight of PEO between 300 K and 600 K was shown to have optimal mechanical and rheological properties for the FDM 3D printing process.

## 5. Characterization of the 3D Printed Pharmaceuticals

Table 4 shows different dosage shapes that have been reported in the literature. 3D printing has the advantage of the ability to design and print dosages of different shapes and sizes to control the release profile. However, in the 3D printing of pharmaceuticals, one of the important parameters is the degree of accuracy between the CAD model and the printed structure. 

**Figure 9:**
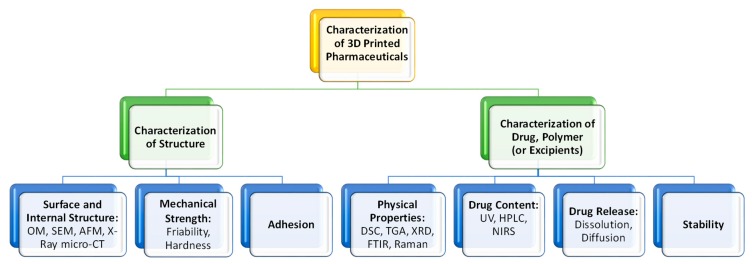
Characterizations required for 3D printed structure, drug, and polymer (or other functional excipients, if necessary).

FDM and PAM 3D printing technologies rely on the extrusion of polymer melts and semi-solids through the nozzle. The major challenge here is to maintain a reproducible and consistent flow of materials throughout the printing process to make the printed dosage form flawless [53]. Typically, surface imperfections are observed in the printed object. Post-treatment method such as the drying method and duration can also affect the structure and properties of the finished products. Several drying conditions (process types, temperature, and duration) are reported for PAM 3D printing. El Aita et al. investigated the drying process and found only 3 h of drying at 200 mbar in a vacuum dryer was sufficient for the complete drying (loss on drying, LOD, <1%) of their printed structure [148]. Other drying conditions reported as using a vacuum dryer at 40 °C for 24 h [18,76,139], on the heated printing platform at 80 °C for 3 h [149], in an oven at 40 °C for 12 h [150,151], left at 20 °C for 12 h [152], in an incubator containing a silica gel bead cartridge at 40 °C for 24 h [63] etc. Drying effect on structure and properties of the finished products are reported as during the drying process shell formation may occur on the outer surface of the tablet which might cause inefficient drying and result in the mechanical properties (friability, hardness) of the tablet [148], deformation of the structure [63,76,150], formation of pores on the surface [152], paste shrinkage [139], etc. The mechanical strength of 3D printed tablets is also important. Materials choices also impact the tensile strength of the tablets. A thorough characterization is required to ensure that the 3D printed structure or dosage form can reliably deliver the right amount of drug in a consistent manner. Figure 9 shows the characterization methods/techniques required for the 3D printed structure, drug, and excipients to ensure the right surface and mechanical strength of the structure as well as drug and excipients’ physical properties and stability maintained.

The surface or cross-section images of the structure can be depicted using optical microscopy (OM), scanning electron microscopy (SEM), and atomic force microscopy (AFM). Images can provide more detailed information about the nature of the surface of the material, such as the presence of pores, cracks, or other inconsistencies [100]. To visualize the internal structure, density, and porosity of the 3D printed structure X-ray Micro Computed Tomography (Micro-CT) can be used [131]. Mechanical tests such as friability and hardness test the strength of a tablet. Ideally, a tablet should be able to remain intact through transportation and storage without breaking or losing material [150]. Adhesion is the interatomic and intermolecular interaction at the interface of two surfaces. Whether the two surfaces adhere well to each other depends on the characteristics of both surfaces at the interface. Interfacial adhesion refers to adhesion between two layers of different compositions. If adhesion is poor between two different polymers, it can lead to brittleness in the final product [153]. Interfacial adhesion is improved when the two different layers have similar properties, such as hydrophilicity [154]. Adhesion can also be achieved by polar functional groups [155]. Adhesion is important when different types of polymers are used in the same structure. Adhesion can be measured by shear tension and torsion tests [156]. Some other tests include a delamination resistance test which measures the force required to separate two layers. A qualitative tape test can be performed, in which an adhesive is attached to one layer and then removed so that the percentage of the layer that becomes detached can be measured [153]. Fourier-transform infrared spectroscopy (FTIR) can be used to examine whether two polymers have chemically bonded to each other, by observing whether certain bonds are present or not. Adhesion can be also be analyzed visually at the interface surface, with a microscope [157].

Tests such as X-ray diffraction (XRD), differential scanning calorimetry (DSC), and FTIR, etc. are used to determine a variety of physical properties. XRD for crystalline, semi-crystalline, and amorphous materials show sharp peaks, a combination of sharp peaks and amorphous halo, and only amorphous halo, respectively. The thermal analysis of the drug or excipient can be done using DSC. Thermogravimetric analysis (TGA) data helps to detect drug degradation [158]. Goyanes et al. determined the degradation of salicylic acid by comparing the weight of the sample with the starting weight at different temperatures [93]. Salicylic acid weight percentage is greatly reduced at temperatures above 140 °C, whereas it completely degrades at about 200 °C [93]. Typically drug content is measured by UV spectrophotometer or high-performance liquid chromatography (HPLC) [60,93]. However, in both methods, the structure needs to be dissolved in a suitable solvent. Vakili et al. have used near infra-red (NIR) hyperspectral imaging technique as a tool to quantify the drug content in 3D printed dosage forms of each printed layer [159]. The tool is currently heavily used in the agricultural and food industries for quality and safety assessments. With future research, NIR hyperspectral imaging can be used as a reliable, rapid and non-destructive method to validate the dosage of printed pharmaceuticals and ensure that the pharmaceuticals are meeting quality standards [159]. Kyobula et al. used Raman spectroscopy to understand the drug distribution on the printed structure [33]. Boetker et al. reported Raman spectroscopy can be considered as a potential PAT (process analytical tool) for the robust production of different 3D printed structures [96]. Dissolution and diffusion tests simulate how the drug would break down in the human body. They are used to determine release profiles and time the drug spends in the body. A drug diffusion test is conducted in vertical glass Franz cells [93] whereas, the dissolution test is done in dissolution tester [18]. The long-term stability can be eliminated by on-demand printing of dosage using 3D printing. However, the dosage should have some short period of stability at least for one or two weeks. Tablet properties such as assay, tensile strength, and dissolution after a short period of stability need to be checked to ensure that the drug does not degrade [67].

## 6. Challenges and Opportunities

### 6.1. Quality and Sterility Aspects

3D printing is well taken in many applications of pharmaceuticals but there still needs improvement of quality and safety aspects of the 3D printed drug products. Several issues such as variation in product qualities (shrinkage, warping, residuals, etc.), mechanical instability, high friability, low drug loading, high post-processing cycles, and an unidentified suitable combination of API and excipient materials need improvement to obtain a quality product in a reproducible manner [5].

Pharmaceutical manufacturing is a regulated and complex process. To meet the requirements of current good manufacturing practice (cGMP), sterilization of manufacturing equipments is essential. Sterility is needed to ensure that the product is contamination free. It should be noted that there is not much mention of sterility in regards to 3D printing of pharmaceuticals. Alomari et al. summarized different methods of the printer cartridge and printer nozzle in inkjet printing of pharmaceuticals [160]. Cheah et al. reported that the parts in SLS printing to be sterile by nature because of the high processing temperature and inert nitrogen-filled fabrication environment [161]. The print head for PAM printing is designed to use disposable tubes that hold the feed material, paste [162]. Several other techniques can be adopted to consider sterility of printings such as the use of removable head, print on surfaces treated with ethanol, print under UV light, etc. [160,163]. More research must be done to get a complete idea of sterilization aspects in FDM and PAM printing of pharmaceuticals.

### 6.2. Regulatory Aspects

The future of 3D printed pharmaceuticals appears to be geared towards the ability of patients to 3D print their necessary pharmaceuticals at home. Printing at home creates a new issue of how to regulate that process. Regulatory issues arise when considering how to ensure that what a patient is printing at home is safe and of adequate quality. In the future, the FDA will have to tackle the problem of how to regulate drug algorithms or drug recipes that individuals could have easy access to at home [164].

The FDA has already approved the 3D printed drug, Spritam^®^ (levetiracetam), in August 2015 for commercial use. There are still numerous issues such as variation in product qualities, mechanical instability, etc. [5] mentioned above that must be improved to completely regulate the 3D printed pharmaceuticals. Alhnan et al. noted that it is likely that the FDA will have to overhaul its traditional regulations in order to adapt to the regulatory needs of 3D printing [53]. The FDA is currently doing its own research on 3D printing and the development of a clear and complete regulatory pathway may take some time [53].

### 6.3. Commercial Manufacturing

Mass manufacturing commercially is always a factor considered when a new technology is developed. However, it is noted that traditional mass manufacturing cannot be replaced by 3D printing, at least in the short term, and it is unlikely to be used for mass production [160]. The use of 3D printing in the fabrication of pharmaceuticals offers major advantages in commercial manufacturing. Combining HME with 3D printing eliminates the need for downstream processing that is present in current pharmaceutical manufacturing practices [165]. FDM printing is known for its use of relatively cheap printers and having minimal to no post-operating processing [62]. The current manufacturing process has limitations related to drug release rates. Drug release rates produced by current manufacturing techniques decrease as a function of time. Ideally, constant release rates are desired, especially for personalized pharmaceuticals. 3D printing enables us to create different polymer matrices that determine tunable release profiles [12]. Using conventional production techniques, such as powder compaction, it is difficult to manufacture the dosage of complex shapes. With 3D printing methods, like FDM or PAM, complex shapes like the torus shape can be created. The torus shape provides increased surface area as well as offers a relatively constant surface area during dissolution [166].

There are some significant challenges in replacing the current commercial manufacturing with 3D printing technology. Compared to current manufacturing processes for drug delivery systems, 3D printing has a low throughput as a result of low processing speeds [167]. Another problem for commercial 3D printing is that all pharmaceuticals manufactured in the United States have to follow the cGMP regulations enforced by the FDA [168]. Melocchi et al. set up a pilot plant that industrialized the FDM 3D printing process. To create the necessary filaments, hot-melt extrusion was carried out using a micro-extrusion manufacturing system. The team paid close attention to the operating temperature and pressure in order to ensure the maintenance of the chemical properties of the starting materials. The group considered non-traditional cooling options because of the water solubility of the polymer. They also designed a prototype FDM 3D printing to follow the cGMP [169].

A few companies are dipping their toes into the 3D printing pharmaceutical industry. Aprecia Pharmaceuticals have already filed a patent application and commercialized Zipdose^®^, a 3D printing system that produces Spritam, a printed medicine. FabRx, a small startup company, is developing and commercializing technology that uses 3D printing to produce personalized pharmaceuticals on-site in pharmacies or clinical trial units. GlaxoSmithKline (GSK) is also looking into the 3D printing of drugs, dedicating a research and development project towards the technology [170].

### 6.4. Personalized Pharmaceuticals

Typically, oral solid dosages are manufactured in pre-defined strength which is determined during early clinical trials based on therapeutic effects observed in the greatest portion of the population [160]. Current approaches to personalized pharmaceuticals involve manually splitting tablets or measuring out specific amounts of liquid dosages. Both of these methods can lead to errors and lack the necessary precision needed for exact personalization [160]. Having the ability to control the dose via 3D printing is a vital tool in the goal to create personalized medicines. Pharmaceuticals can be individualized for each patient by controlling the dose.

**Figure 10:**
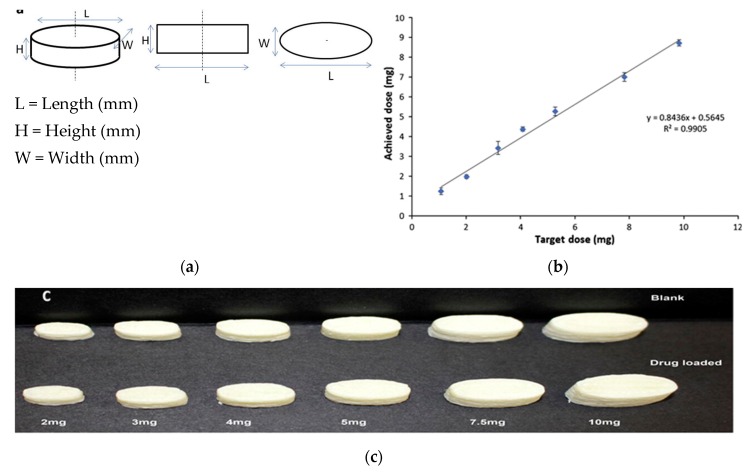
(**a**) Design of tablets, (**b**) the relationship between the target and achieved prednisolone dose, and (**c**) PVA based FDM 3D printed tablets having different dose strengths [56].

Skowyra et al. investigated how the FDM 3D printing process can be used to control the dose of the active ingredient by changing the printed volume using computer software [56]. Skowyra et al. compared the theoretical doses determined by tablet mass with HPLC measured doses of prednisolone, a model pharmaceutical drug, to establish a linear correlation between the parameters. This linear relationship is used to determine an equation that calculates the required dimension needed to achieve the target dosage. Results show (Figure 10) a strong correlation between the target dose and the measured dose proving the possibility of controlling the dose using FDM 3D printing via volume manipulation and make it suitable for personalized pharmaceuticals development [56].

Target or theoretical dose D (mg) = M.S/100

The mass of the printed tablet M (mg) = 1.0322 V + 24.898

The volume of the design V (mm^3^) = L × W × H = 0.04 × *π* × L^3^ (Consider ellipse shape and W = H = 0.4L)

The filament loading percentage S = 100× (Mass of drug/Total mass of filament)

The required dimension (L) to achieve a target dose (D) from the filament with loading percentage (S) can be calculated as

(5) $$L=\sqrt[{3}]{25\frac{\left(\frac{1000}{S}\right)-24.898}{1.0322}}$$

### 6.5. Medication Adherence and Multi-Drug Printing

Medication adherence, or taking drugs correctly, is usually defined as the extent to which patients take drugs as prescribed by the doctor [171]. A drug that is not taken or taken at the wrong intervals, in under-dose or over-dose defeats its intended efficacy to patient’s disease treatment [172]. The difficulty of tracking with multiple drugs, complex dosing regimen, and swallowing difficulties commonly causes poor adherence [173]. Poor adherence can interfere with the proper treatment of diseases and aggravate the complications. One of the approaches to improve poor adherence is by incorporating multiple drugs in the same matrix and tuning their release profile in a controlled manner. This is also known as poly-pharmacy or polypill [174].

To the extent of the authors’ knowledge, there is only one commercially available multidrug pill named Polycap^TM^, manufactured by Cadila Pharmaceuticals Ltd. India [175,176]. It is a five-drug capsule that is used to treat cardiovascular disease and stroke. Current manufacturing practices provide limitations to the successful creation of multi-drug pills [18]. Traditional direct compression manufacturing processes is unable to make multi-drug pills due to challenges of drug processing without functional excipients, limited capacity to compress multiple bulk materials having different drugs, and eventually control the pill size to make it swallowable. In current practices, manufacturers are only able to control release profiles by using a different material or polymer during fabrication [165]. With the advent of 3D printing, drug makers have the ability to formulate pharmaceuticals that have a complex design and fabricate multi-drug pills. 3D printing has made it possible by the creation of different infill densities and patterns that would be an advantage in multi-drug printing. Varying the infills provides a method to control the release profiles [146].

Recently, there have been several publications that reported multi-drug printing using 3D printing [18,74,76,177,178,179,180,181]. In 2015, Khaled et al. used PAM printing to create a multi-drug pill with five drugs and two different release profiles [18]. Melocchi et al. designed a hollow multidrug capsule that is made up of two separate compartments connected by a joint [169]. Goyanes et al. used FDM 3D printing to construct a multilayer pill with alternating layers of two different drugs [108]. With these attempts, researchers are seeking solutions to different problems related to the fabrication of multi-drug pills. Goyanes et al. observed that the layers of the fabricated multi-drug pill are difficult to separate because they adhere independently of the type of layers during the printing process. This addresses a current problem in current bilayer technology where insufficient layer bonding and bilayer hardness damage the products of the technology [182]. One problem that still needs to be addressed is the size of the multi-drug pills. Incidentally, a multi-drug pill with multiple release capabilities often requires large amounts of excipients which can make the pill too large for swallowing [11]. There is still room for improvement to decrease the size of the multi-drug pill for easy swallowing, control drug release, tune the dose strength, etc.

## 7. Conclusions and Outlook

3D printing of pharmaceuticals is an emerging technique that is especially advantageous for personalized medicine, offers design flexibility, high complexity, on-demand, and cost-effective production. With an exceptionally high degree of control and flexibility, 3D printing technology opens up the possibility of producing any types of pharmaceutical formulations, dosages and tailor-made drug delivery systems. Despite the last decade has seen massive achievements in other manufacturing industries, such as aerospace and automobile, 3D printing of pharmaceuticals is still at its infancy and its potential yet to be fully explored. Various technical and regulatory challenges need to be overcome to fulfill its real potential in the pharmaceutical industry. Though a large scale production of pharmaceuticals using 3D printing might be a long way from now, personalized medicine is possible in-house for immediate use. Future work to enable drug product manufacture using FDM and PAM 3D printing technologies should include the suitability and characterization of polymers and other excipients amenable to processing. Polymer materials and their properties, specifically their rheology should be investigated to allow a wider formulation and 3D printing design space. A better understanding of the rheological properties of API–polymer mixtures and their measurement is necessary for the successful 3D printing of pharmaceuticals. This review provides a holistic materials–processing perspective for the extrusion-based 3D printing technologies which provides a guideline in selecting suitable polymers for FDM and PAM 3D printing processes, and also the proper characterization techniques for printed structures, drugs, and excipients. The authors hope that the insights of this review will provide a useful stimulus to encourage future research into the optimization of the pharmaceutical extrusion-based 3D printing process.

## Figures and Tables

**Table 1:** Comparison of FDM and PAM 3D printing technologies.

Technology	FDM 3D Printing	PAM 3D Printing
Advantages	Low-cost printing technology. No post-processing is required. Better drug uniformity.	○ Works at room temperature. ○ High drug loading is achieved. ○ Suitable for multi-drug pill (polypill) printing.
Limitations	High-temperature processing is required which is not suitable for thermally labile drugs. Pre-processing steps of filament making are required. Lack of suitable biocompatible/biodegradable thermoplastic polymers. Active pharmaceutical ingredient (API) degradation may occur due to the high processing temperature.	○ Post-processing, drying, is required. ○ Polymer rheological properties impact on structure formation and printing process. ○ Printing resolution is depended on nozzle size. ○ Toxicity and drug instability may occur due to the usage of organic solvents.

**Table 2:** Examples of material compositions used in extrusion-based 3D printing.

Extrusion Method	Materials Composition	Drug Release Type	References
FDM	95% Polyvinyl alcohol (PVA), 5% drug (Paracetamol)	Controlled Release	[55]
	90–100% Hydroxypropyl cellulose (HPC), 2–10% Poly (ethylene glycol) (PEG), 2% drug (acetaminophen)	Pulsatile Release	[70]
	45.5% Hydroxypropyl methylcellulose (HPMC E5) and 19.5% Ethylcellulose (EC) or HPC, 30% drug Acetaminophen (APAP), 5% Kollidon	Controlled Release	[71]
	45% HPC, 50% drug (Theophylline), 5% triaceten	Immediate Release	[72]
	65–90% PVA, 10–35% drug (Ciprofloxacin hydrochloride), 2% dibutyl sebacate	Controlled Release	[73]
	60.35% PVA, drugs = 5% Lisinopril dihydrate, 2.5% Amlodipine besylate, 1.25% indapamide, 5% rosuvastatin calcium. 25.9% sorbitol	Various (Depends on Drug)	[74]
	60% HPMC, 15% Eudragit, 20% drug (Carvedilol), 5% D-α-Tocopheryl polyethylene glycol 1000 succinate (TPGS)	Extended Release	[75]
PAM	2% HPMC, 81% drug (Guaifenesin), 7% Sodium starch glycolate (SSG), 10% Microcrystalline cellulose (MCC)	Controlled Release	[17]
	7.1% HPMC, 3.5% drug (Glipizide), 17.8% PEG, 25% tromethamine, 46.6% lactose	Sustained Release	[76]
	72.1% 2-Hydroxypropyl-β-cyclodextrin (HPβCD), 2.4% HPMC, 24% drug (Carbamazepine)	Immediate Release	[77]
	2% Carbopol, 35% drug (Diclofenac sodium), 20% Lactose, 5% Polyplasdone, 21% Avicel PH101, 14% Avicel PH105	Modified Release	[60]

**Table 3:** Application of rheological techniques for extrusion-based (FDM, PAM) 3D printing and the used API-excipient combinations; literature examples.

Rheological Techniques; Application	Excipients (Polymers, Plasticizers, Other)	APIs	Reference
**FDM 3D Printing**			
Oscillatory shear; controlling the dosage forms	Eudragit EPO, Tri-calcium phosphate (TCP), triethyl citrate (TEC)	Enalapril maleate (EM) and hydrochlorothiazide (HCT)	[132]
Oscillatory shear; effect of excipient content on the flow properties and API release	PLA, Hydroxypropyl methylcellulose (Metolose^®^)	Nitrofurantoin	[96]
Oscillatory shear; evaluation of materials for FDM printability and process modulation	Hydroxypropyl methylcellulose (HPMC) Affinisol HME 15LV, Kollidon SR [a mixture of insoluble poly(vinyl acetate) (PVAc) and soluble povidone (PVP)], Eudragit EPO, hydroxypropyl cellulose (HPC) SSL, Kolliphor TPGS	Carvedilo l	[136]
Oscillatory shear; effect of polymer molecular weights on the flow properties and FDM printability	PEO, PEG	Theophylline	[135]
Steady-state (zero-shear) viscosity and Oscillatory shear; API–polymer miscibility, assessment of FDM 3D printability	Poly(ε-caprolactone)	Indomethacin	[134]
Oscillatory shear; drug-polymer, polymer–polymer, and drug–polymer–polymer miscibility, and evaluation of polymers or polymer blends for FDM 3D printability and drug release	Polyvinylpyrrolidone-vinyl acetate copolymer (Kollidon^®^ VA64), polyvinyl alcohol-polyethylene glycol graft copolymer (Kollicoat^®^ IR), Hydroxypropyl Methylcellulose (HPMC), hydroxypropyl methylcellulose acetate succinate (HPMCAS)	Haloperidol	[137]
Steady-state (zero-shear) viscosity and Oscillatory shear; drug-polymer miscibility, effects of particle morphological changes in the drug-polymer mixture on the flow behaviours	Polyethylene oxide (PEO), methacrylate copolymer (Eudragit^®^ E PO)	Paracetamol and ibuprofen	[138]
Oscillatory shear; effect of non-melting filler on FDM 3D printing quality and drug release	Methacrylic polymer (Eudragit EPO), tri-calcium phosphate (TCP)	5-aminosalicylic acid (5-ASA), captopril, theophylline, and prednisolone	[62]
Oscillatory shear; effects of plasticizer on processing parameters of FDM 3D printing	Polycaprolactone, poly- (ethylene glycol) (PEG, M_w_ = 200, 4000 and 8000 g/mol)	Ciprofloxacin	[116]
PAM 3D Printing			
Creep recovery, cross-over modulus; probe the viscoelastic properties of paste	Carbopol (CP-794), Avicel PH101 and PH105, Polyplasdone, and glycerol	Diclofenac Sodium	[139]
Rheogram (plot of shear stress vs. shear rate); appropriate extrusion of paste and 3D printability	Hydroxypropyl methylcellulose (HPMC 2208 type), Crospovidone (Kollidon CL-F), D-Mannitol, and Polyethylene glycol (PEG) 4000	Naftopidil	[63]

**Table 4:** Pharmaceutical dosages of different shapes or forms, sizes and complexities that had been 3D printed and reported in the literature.

Dosage Shape, Size, Complexity		References
**FDM 3D Printing**		
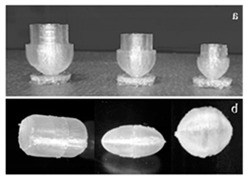	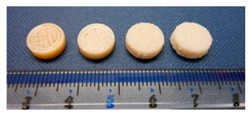	[70,142]
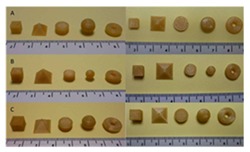	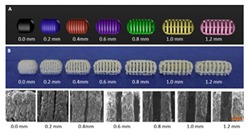	[55,72]
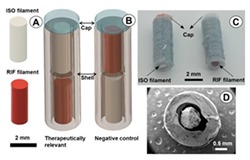	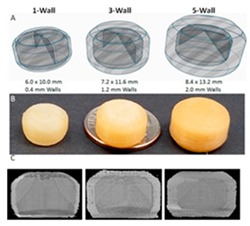	[143,144]
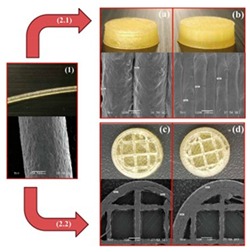	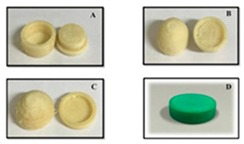	[75,145]
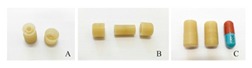	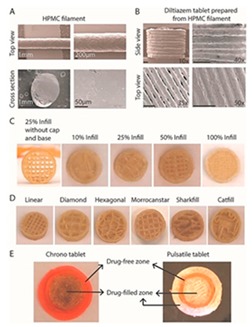	[103,146]
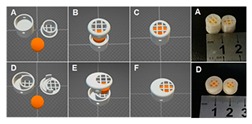	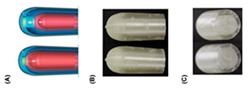	[99,147]
**PAM 3D Printing**		
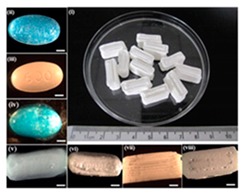	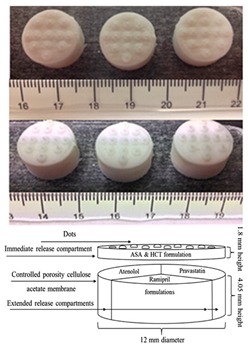	[17,18]
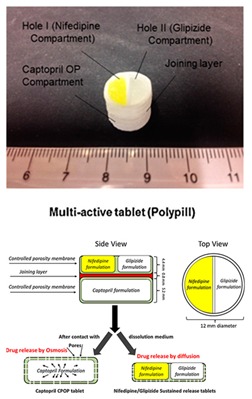	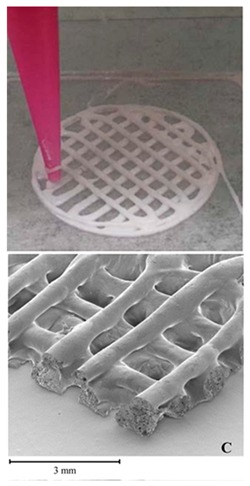	[76,77]
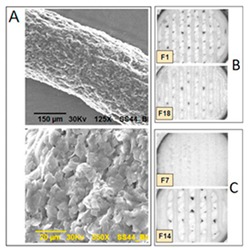		[60]
